# When more diagnoses do not mean more disease: a data-driven reassessment of global chronic disease trends, 2011–2025

**DOI:** 10.7189/jogh.16.04214

**Published:** 2026-06-12

**Authors:** Marcin M. Nowak, Leszek Pączek

**Affiliations:** 1 Department of Cardiology and Internal Diseases, Międzyleski Specialist Hospital in Warsaw, Warsaw, Poland; 2 Department of Clinical Immunology, Medical University of Warsaw, Warsaw, Poland; 3 Institute of Biochemistry and Biophysics, Polish Academy of Sciences, Warsaw, Poland

## Abstract

**Background:**

Rising global numbers of chronic noncommunicable diseases (NCDs) are commonly interpreted as evidence of a growing epidemic. In 2011, we hypothesised that this perception is partly driven by population ageing, expanding diagnostic criteria, and improved detection rather than a uniform increase in underlying biological risk. This study reassesses that hypothesis using contemporary global data.

**Methods:**

We conducted a descriptive, comparative epidemiological analysis using publicly available data sets from the Global Burden of Disease, World Health Organization Global Health Estimates, and the International Diabetes Federation. Absolute case counts and deaths were analysed alongside age-standardised mortality rates to distinguish demographic effects (population growth and ageing), diagnostic expansion, and changes in underlying risk. Trends were evaluated relative to 1990 and 2011 baselines.

**Results:**

Absolute numbers of cases and deaths from major NCDs have continued to rise globally, largely reflecting population growth and ageing. In contrast, age-standardised mortality rates have declined substantially for cardiovascular disease and chronic obstructive pulmonary disease and have stabilised for other conditions. This divergence between increasing absolute burden and stable or declining age-specific risk is consistent across major diseases. Expanded diagnostic criteria, improved detection, and increased survival have further contributed to rising prevalence, particularly in older populations.

**Conclusions:**

Rising absolute counts of NCDs are largely explained by demographic change and diagnostic expansion, while age-standardised trends suggest stable or declining risk for several major conditions. These findings support a more nuanced interpretation of global chronic disease trends, integrating demographic, diagnostic, and risk-factor perspectives. Careful use of age-standardised measures alongside absolute counts is essential for accurate monitoring and for informing public health priorities.

Noncommunicable diseases (NCDs) are the leading cause of mortality worldwide, accounting for approximately three-quarters of all global deaths according to recent estimates from the World Health Organization (WHO) and the Global Burden of Disease (GBD) study. Over recent decades, the absolute number of individuals living with chronic conditions, including cardiovascular disease (CVD), diabetes (DM), chronic kidney disease (CKD), and chronic respiratory diseases (COPD), has increased substantially. This trend is widely interpreted as evidence of a growing global epidemic of chronic disease.

Previous analyses, including our earlier work from 2011, have suggested that the apparent increase in chronic disease burden may be partly explained by demographic changes, improved survival, and expanding diagnostic criteria rather than a uniform increase in biological risk [1]. We argued that much of the increase in case numbers resulted from broader and more sensitive diagnostic criteria, improved detection and screening, and population ageing. Accordingly, we cautioned that direct comparisons between contemporary and historical disease statistics may be misleading, and that the concept of an ‘epidemic’ should be interpreted with caution.

Chronic kidney disease illustrates the complexity of applying diagnostic thresholds in population-based analyses. Clinically, CKD is defined by either reduced glomerular filtration rate (eGFR<60 mL/min/1.73 m^2^) or evidence of kidney damage, most assessed by albuminuria (ACR), persisting for at least three months. However, in large epidemiological data sets, CKD prevalence is often estimated primarily using eGFR thresholds for reasons of feasibility and comparability. This approach may overestimate disease burden in older populations, where age-related decline in eGFR is common, highlighting the importance of cautious interpretation of such data [2,3].

Overdiagnosis, defined as the diagnosis of a condition that would not cause symptoms or harm if left untreated, has emerged as an important concept in modern medicine. It is particularly relevant in ageing populations, where physiological changes may meet diagnostic thresholds without corresponding clinical impact. In such contexts, increased detection may inflate prevalence estimates without reflecting a proportional increase in clinically meaningful disease.

Although diagnostic precision has improved, current definitions do not always account for the physiological effects of ageing. In Poland, for example, approximately 2.5 million adults live with diabetes, most of them over the age of 60 [4]. Despite this, individuals older than 65, who constitute the majority of patients with chronic diseases, remain underrepresented in pivotal clinical trials. Recent analyses indicate that only about 30% of trial participants are aged ≥65, with very limited inclusion of individuals above 75 years, even in oncology [5–7]. These observations highlight the challenges of applying uniform diagnostic thresholds across heterogeneous populations. While age-related physiological changes may increase the likelihood of meeting certain diagnostic criteria, this does not diminish the clinical relevance of these conditions. Rather, it underscores the importance of interpreting such findings in the context of overall risk, functional status, and potential benefit from intervention.

Importantly, this study does not question the clinical validity of these conditions but instead aims to improve the interpretation of their epidemiological trends at the population level.

This study therefore aims to reassess whether the apparent global ‘epidemic’ of noncommunicable diseases reflects a true biological increase in disease risk or is predominantly driven by demographic and diagnostic dynamics, updating the original 2011 hypothesis using contemporary data from 2021–2025.

Despite the availability of increasingly detailed global data sets, the relative contributions of demographic change, diagnostic expansion, and evolving risk-factor exposure to observed trends remain incompletely resolved.

Importantly, this study does not propose redefining disease based on prevalence. Rather, it emphasises that epidemiological trends should be interpreted in the context of underlying risk, clinical relevance, and population structure.

## METHODS

This study reassessed global trends in four major noncommunicable diseases: cardiovascular disease, diabetes mellitus, chronic kidney disease and chronic obstructive pulmonary disease, following the original conceptual framework distinguishing true biological change from apparent change driven by diagnostic expansion, improved detection, and population ageing.

The analysis was based on a descriptive, comparative assessment of publicly available global health data sets. Primary data sources included the Global Burden of Disease 2021–2025 study, which provides estimates of incidence, prevalence, mortality, and age-standardised rates for 204 countries and territories. Complementary data were obtained from the WHO Global Health Estimates (2024–2025) and the International Diabetes Federation (IDF) Diabetes Atlas (2024). Additional contextual information was drawn from Our World in Data and recent clinical guideline updates, including GOLD 2024 for COPD, the CKD-EPI 2021 equation for CKD, and WHO cardiovascular and diabetes guidance.

### Data analysis

A comparative analytical framework was applied to contrast absolute case counts and deaths with age-standardised mortality rates (ASMRs). Absolute counts were interpreted primarily as reflecting demographic changes, including population growth and ageing, as well as diagnostic expansion. In contrast, ASMRs were used as indicators of underlying age-specific risk. Trends were evaluated relative to baseline years (1990 and 2011), and percentage changes over time were calculated. Where applicable, analyses considered variation across income groups and sociodemographic index (SDI) categories.

All data sets were harmonised to standard population structures as defined by the original sources (GBD/WHO). Age-standardised rates were used to adjust for differences in population age distribution across time and regions, enabling comparison of underlying risk independent of demographic change.

Special attention was given to evolving diagnostic definitions and thresholds, including high-sensitivity troponin assays for myocardial infarction, HbA1c criteria for diabetes, eGFR-based definitions for CKD, and the fixed FEV_1_/FVC ratio for COPD. These changes were reviewed to assess their potential impact on reported prevalence and to distinguish epidemiological trends related to true disease dynamics from those influenced by diagnostic practices.

In the case of CKD, it is acknowledged that epidemiological estimates are often based on eGFR thresholds alone, whereas clinical diagnosis additionally requires markers of kidney damage, such as albuminuria. This discrepancy was considered in the interpretation of prevalence estimates.

No inferential statistical modelling was performed, as the objective of the study was not hypothesis testing but structured comparison of established epidemiological indicators. The analysis combined quantitative components – comparison of absolute counts, percentage changes, and ASMR trends – with qualitative assessment of changes in diagnostic criteria and screening practices.

Uncertainty intervals were not recalculated but were interpreted as reported in the original data sets. Accordingly, all estimates, particularly projections, are subject to inherent uncertainty related to demographic assumptions, data quality, and future risk-factor trajectories.

The analysis focused on global aggregate trends. Regional heterogeneity and within-country variations were not formally modelled but are recognised as important sources of variation and are considered in the interpretation of findings.

## RESULTS

Results are presented as descriptive observations based on reported epidemiological estimates, with interpretation of underlying mechanisms addressed in Discussion. Key differences between 2011 context and contemporary evidence (2021–2025) are summarised in **Table 1**.

**Table 1:** Global comparison Table (2011–2025) summarising how reality evolved *vs*. what we discussed in the original article

Disease / topic	2011 context	2021–2025 data	Change/interpretation
Global life expectancy	≈ 65 y globally (2005 data); projected 85 y in richest countries by 2030.	2023 avg = 73.2 y globally (Our World in Data 2024) [8,9].	+8 y since 2005. Life expectancy continues rising but at slower pace due to NCDs + COVID setbacks.
Cardiovascular disease (CVD)	‘29% of deaths’; expected to become top cause worldwide by 2020.	2021: 19.4 million deaths, ≈ 32% of all NCD deaths. Age-standardised mortality has fallen ≈ 34% since 1990 [5].	Projection came true – CVD = #1 killer, but relative risk per age group falling. Prevention and therapy advance effective.
Diabetes mellitus (DM)	285 million cases (6.4% adults); projected 439 million by 2030.	≈ 589 million in 2024, projected 853 million by 2050 (IDF 2024). 3.4 m deaths / yr; ≈ 43% undiagnosed [6,7].	Far exceeded 2011 projection and reflects a combined effect of increasing metabolic risk, demographic change, and improved detection.
Chronic kidney disease (CKD)	≈ 1.1 million on dialysis; criteria (eGFR<60 mL/min) may over-diagnose elderly.	≈ 700–800 million cases ( ≈ 10% adults); >3 million deaths / yr. Age-standardised rates stable; absolute numbers [10,11].	Caution on diagnostic inflation is valid, but CKD is now confirmed as a major global NCD driver linked to DM & CVD.
Chronic obstructive pulmonary disease (COPD)	2.75 million deaths (4.8% global mortality); projected #3 cause by 2020.	2023: 3.2 million deaths, still ≈ 3rd global cause (WHO 2024); >85% in LMICs. Pollution + biomass > smoking in many regions [12].	Projection accurate; burden shifted toward environmental/household pollution rather than smoking alone.
Main diagnostic / classification issue	Warned that changing definitions (troponin for MI, lower glucose cut-offs, eGFR use, fixed FEV_1_/FVC ratio) inflate prevalence and hinder comparisons.	Indeed – diagnostic and screening updates (2018 MI definition, CKD-EPI 2021, GOLD 2024, HbA1c criteria) broaden detection. Modern GBD uses age-standardisation to correct for this.	These methodological considerations are now widely reflected in modern epidemiological practice.
Public-health focus	NCD ‘epidemic’ concept questioned; called for evidence-based comparison tools.	WHO & UN now treat NCDs as top priority (75% of global deaths). Explicit targets to reduce premature NCD mortality by 33% by 2030 (SDG 3.4).	NCDs confirmed as central to global health; policy now aligns with 2011 concerns about measurement and focus.

NCDs – noncommunicable diseases, CVD – cardiovascular disease, DM – diabetes mellitus, CKD – chronic kidney disease, COPD – chronic obstructive pulmonary disease

**Figure 1:**
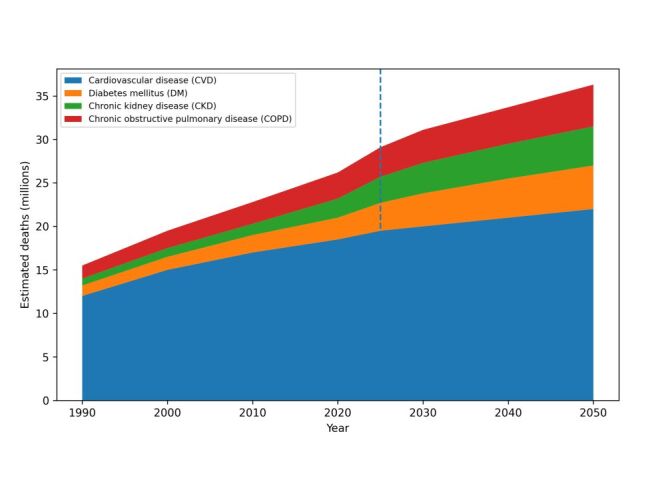
Global deaths from major noncommunicable diseases (CVD, DM, CKD, COPD), 1990–2050. Stacked area chart showing estimated deaths by cause. CVD remains the largest contributor throughout the period, while DM and CKD show progressively increasing contributions. COPD demonstrates a steady but more moderate rise. The vertical dashed line indicates the transition between observed data (1990–2025) and projected estimates (2025–2050). The figure illustrates a continuous increase in absolute mortality, largely reflecting population growth and ageing. CVD – cardiovascular disease, DM – diabetes mellitus, CKD – chronic kidney disease, COPD – chronic obstructive pulmonary disease.

Since 2011, the quality, scope, and comparability of global health data have improved substantially. The Global Burden of Disease 2021 study provides detailed epidemiological estimates across 204 countries and territories [10]. In 2021, approximately 673 million people were living with chronic kidney disease (CKD) worldwide [11]. According to WHO estimates, noncommunicable diseases account for approximately 75% of global deaths – around 43 million annually – with nearly 19 million attributable to cardiovascular disease [8,12] (**Figure 1**). Projections should be interpreted with caution due to uncertainty related to demographic assumptions and future risk-factor trajectories.

**Figure 2:**
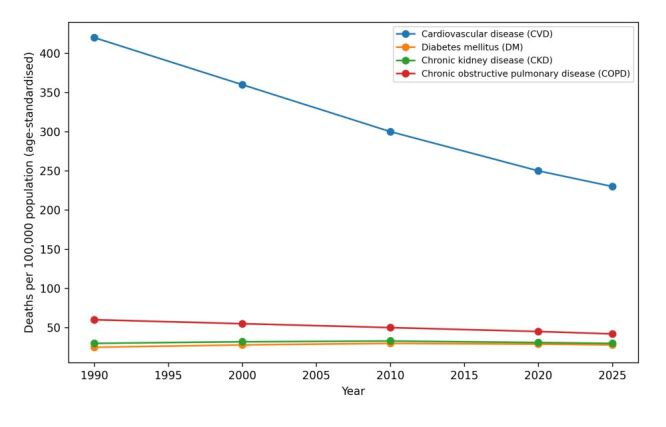
Age-standardised global mortality rates for the same conditions, 1990–2025. Age-standardised mortality rates (ASMRs) are expressed per 100 000 population and adjust for differences in population age structure over time. Declining trends, particularly for CVD and COPD, indicate reduced age-specific mortality despite increasing absolute numbers of deaths. CVD – cardiovascular disease, COPD – chronic obstructive pulmonary disease.

Across major NCDs, a consistent pattern is observed: absolute numbers of cases and deaths have increased over time, while age-standardised mortality rates have declined or stabilised. For example, although the total number of CVD-related deaths has risen since 1990, the global ASMR has decreased by approximately one-third [9] (**Figure 2**). This divergence between absolute counts and age-standardised rates is consistently observed across multiple conditions.

In 2021, NCDs accounted for at least 43 million deaths globally, including approximately 18 million premature deaths occurring before the age of 70 [8,12]. Most of these deaths occurred in low- and middle-income countries (LMICs). While absolute case counts and mortality continue to rise, many age-standardised rates have plateaued or declined.

Cardiovascular disease remains the leading global cause of death, responsible for approximately 19.4 million deaths in 2021, compared with 12.3 million in 1990 [9]. Despite this increase in absolute numbers, the global age-standardised mortality rate has declined by roughly 34%, with regional variation across countries.

Diabetes mellitus has shown a marked increase in global burden. Among adults aged 20–79, prevalence reached approximately 589 million in 2024 and is projected to rise to 853 million by 2050. Nearly half of all cases remain undiagnosed, and the burden is disproportionately concentrated in LMICs. Estimated diabetes-related deaths reached approximately 3.4 million in 2024 [13,14].

Chronic kidney disease affects an estimated 700–800 million individuals globally, representing more than 10% of the adult population [11,15]. Age-standardised rates have remained relatively stable, while absolute numbers have increased.

Chronic obstructive pulmonary disease remains a major contributor to global mortality and morbidity. According to WHO estimates, more than 85% of cases occur in LMICs. A substantial proportion of the global burden is attributable to non-smoking exposures, including ambient air pollution, household biomass fuel use, and occupational factors [16].

Recent GBD analyses provide consistent age-standardised trends across countries, allowing improved comparability over time [10]. Across conditions, increases in absolute case counts and deaths occur alongside stable or declining age-standardised mortality rates.

**Figure 3:**
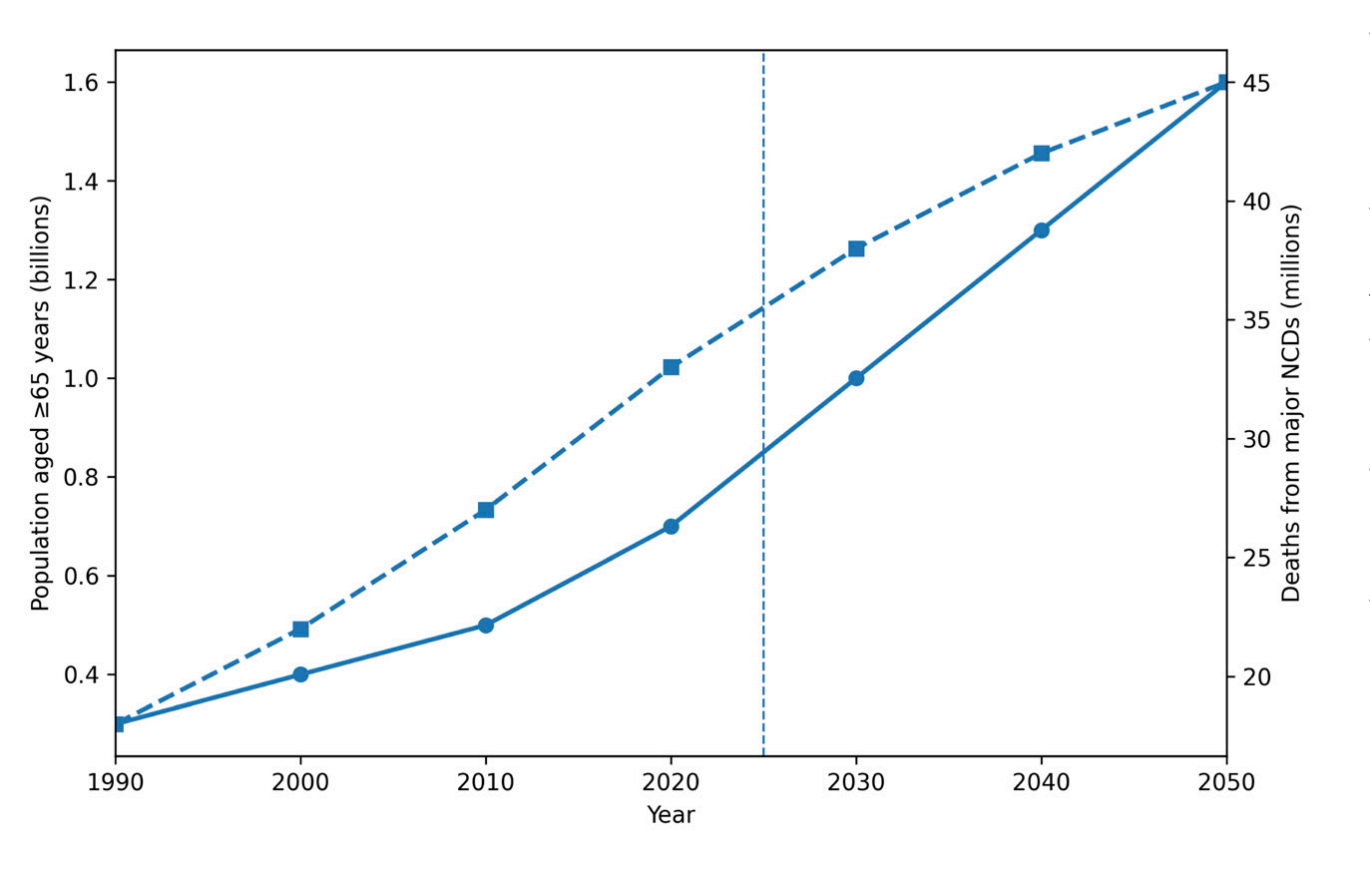
Global population ageing and total deaths from major noncommunicable diseases (NCDs), 1990–2050. The solid line represents the global population aged ≥65 years (left axis, billions), while the dashed line shows total deaths from major NCDs (right axis, millions). The vertical dashed line indicates the transition between observed data (1990–2025) and projected estimates (2025–2050). The parallel increase highlights the contribution of demographic ageing to rising absolute NCD mortality. Projections should be interpreted with caution due to uncertainty in demographic and epidemiological assumptions. Data sources: United Nations World Population Prospects 2024 [17]; IHME GBD 2021–2024 [10,18]; WHO Global Health Estimates 2024 [8,12]. NCDs – noncommunicable diseases.

Population ageing closely parallels these trends. Between 1990 and 2025, the number of individuals aged ≥65 years increased substantially, contributing to rising absolute numbers of NCD cases and deaths (**Figure 3**). When adjusted for population structure, mortality rates decline across age groups.

In parallel, changes in diagnostic definitions and screening practices have expanded disease detection over time. Updates in clinical criteria and the adoption of more sensitive diagnostic tools have influenced reported prevalence across all major NCD categories.

At the same time, global patterns of risk exposure have evolved. Metabolic risk factors, including elevated body mass index, high blood glucose, and hypertension, as well as environmental exposures such as air pollution, now contribute substantially to disease burden worldwide.

Overall, the observed trends indicate increasing absolute burden of major NCDs alongside stable or declining age-standardised mortality rates across multiple conditions.

The present findings support key elements of the 2011 hypothesis, indicating that diagnostic expansion and demographic change account for a substantial proportion of the observed increase in noncommunicable disease burden. At the same time, the absolute global burden of these conditions has increased markedly and now represents a central component of 21st-century global health. Cardiovascular disease remains the leading cause of death worldwide, while diabetes and chronic kidney disease contribute an increasingly large share of the burden, and chronic obstructive pulmonary disease continues to impose a substantial impact with evolving risk profiles. These observations underscore the importance of interpreting epidemiological trends using age-standardised measures alongside absolute counts.

Despite increasing total numbers of deaths, age-standardised mortality has declined – particularly for cardiovascular disease and chronic obstructive pulmonary disease – reflecting improvements in prevention, treatment, and, to some extent, changes in diagnostic practices.

A consistent divergence is observed between rising absolute counts and declining age-standardised mortality rates, indicating that multiple factors contribute to current trends. These include demographic changes, evolving diagnostic practices, and shifts in risk-factor exposure.

Since 1990, age-standardised mortality rates for major NCDs have declined by approximately 25–40%, while absolute death counts have continued to increase. This pattern reflects the combined effects of longer life expectancy, population ageing, and expanded diagnostic criteria.

The rise in absolute NCD mortality closely parallels global population ageing [8,10,12,17-19] (**Figure 3**). Between 1990 and 2025, the number of individuals aged ≥65 years increased substantially, accounting for much of the observed growth in chronic disease mortality. When adjusted for population structure, mortality risk within age groups declines, suggesting that demographic expansion, rather than a uniform increase in individual disease risk, plays a dominant role in shaping these trends.

Projections to 2050 indicate that this pattern is likely to persist. Although the global population will continue to age, age-standardised mortality rates for major NCDs are expected to stabilise or decline modestly [8,10,12,18].

The proportion of adults ≥65 years rose from ~ 6% ( ≈ 320 million) in 1990 to ~ 11% ( ≈ 870 million) in 2025 and is projected to exceed 16% ( ≈ 1.6 billion) by 2050 [17,19]. Over the same period, annual deaths from the four leading NCDs (CVD, DM, CKD, COPD) rose from ≈ 18 million to ≈ 38–40 million, while age-standardised mortality declined by 25–40%. This parallel increase demonstrates that population growth and ageing are the dominant drivers of rising absolute disease burden.

## DISCUSSION

This study provides a structured epidemiological interpretation of global chronic disease trends rather than a causal or inferential statistical analysis. It offers a databased reassessment of contemporary patterns, distinguishing between absolute disease burden and age-specific risk.

The findings presented in the Results section consistently demonstrate a divergence between increasing absolute disease burden and stable or declining age-standardised mortality rates across major noncommunicable diseases. This distinction is fundamental, as absolute counts largely reflect population growth and ageing, whereas age-standardised measures provide insight into underlying risk.

The results indicate that increases in absolute numbers of NCD cases are predominantly driven by demographic change, while age-standardised mortality trends suggest stabilisation or decline for several major conditions. Importantly, these findings should not be interpreted as evidence that chronic diseases are diminishing in importance. Rather, they underscore the need for careful interpretation of epidemiological metrics in ageing populations.

Diabetes represents an important exception to this overall pattern. Unlike cardiovascular disease and chronic obstructive pulmonary disease, its increasing prevalence reflects not only demographic and diagnostic influences but also a substantial rise in underlying risk, driven primarily by obesity, sedentary behaviour, and broader metabolic changes. While demographic and diagnostic factors remain relevant, the magnitude of the observed increase suggests that changes in risk-factor exposure play a dominant role. This distinction is essential, as it demonstrates that disease-specific trends may diverge from general patterns and cannot be explained by a single mechanism.

Risk-factor dynamics therefore play a critical role in shaping global disease patterns and must be considered alongside demographic and diagnostic influences. The global rise in diabetes is closely linked to increasing prevalence of obesity, sedentary lifestyles, and dietary changes. Similarly, in COPD, a growing proportion of disease burden is attributable to non-smoking exposures, including ambient air pollution, household biomass fuel use, and occupational factors, particularly in low- and middle-income countries. These observations highlight the importance of integrating risk-factor trajectories into the interpretation of epidemiological trends.

Overdiagnosis provides an additional interpretative framework for understanding these findings. Defined as the diagnosis of a condition that would not cause symptoms or harm if left untreated, overdiagnosis is particularly relevant in ageing populations, where physiological changes may overlap with diagnostic thresholds. In such contexts, expanded detection may increase recorded prevalence without a proportional increase in clinically meaningful disease. This does not undermine the validity of diagnosis but rather emphasises the need to align epidemiological interpretation with clinical relevance.

At the same time, increased prevalence of chronic conditions in older populations does not diminish their clinical importance. Many of these conditions are associated with significant risks of adverse outcomes and require appropriate management. The present analysis does not question the validity of these diagnoses but highlights that, at the population level, rising prevalence reflects a combination of demographic ageing, cumulative lifetime exposure to risk factors, and changes in detection. Distinguishing these components is essential for accurate interpretation without altering clinical decision-making.

Multimorbidity and competing risks further complicate the interpretation of cause-specific mortality trends. As populations age, individuals increasingly experience multiple coexisting chronic conditions, and improvements in survival from one disease may shift mortality toward others. These dynamics can influence observed mortality patterns without reflecting changes in underlying disease risk, reinforcing the importance of age-standardised measures.

This study has several limitations. It is based on secondary data and does not include primary statistical modelling. Additionally, variations in case definitions, data quality, and reporting across data sets may affect comparability. Risk-factor trends were not quantitatively modelled, and regional heterogeneity was not formally analysed.

Overall, these findings support a more nuanced and structured interpretation of global chronic disease trends, integrating demographic, diagnostic, and risk-factor perspectives within a single analytical framework. The divergence between rising absolute counts and stable or declining age-standardised mortality observed in this study is consistent with patterns expected in the presence of both demographic change and potential overdiagnosis.

It is important to distinguish between the definition of disease at the individual level and its interpretation at the population level. A condition may remain clinically valid regardless of its prevalence, particularly if associated with adverse outcomes. However, when highly prevalent conditions are assessed at the population level, especially in ageing societies, their increasing frequency may reflect demographic structure as much as changes in underlying risk. This distinction is central to interpreting global health trends without undermining clinical decision-making.

This study does not evaluate the appropriateness of specific diagnostic criteria but highlights how changes in detection and classification influence the interpretation of population-level trends. Accordingly, the findings should be interpreted as descriptive and hypothesis-generating rather than causal.

The novelty of this study lies in the integrated interpretation of demographic, diagnostic, and risk-factor dynamics across multiple diseases using contemporary global data.

## CONCLUSIONS

This study provides a structured, databased reinterpretation of global chronic disease trends, integrating demographic, diagnostic, and risk-factor perspectives. Several key conclusions emerge.

First, the central hypothesis proposed in 2011 is supported by current evidence: a substantial proportion of the observed increase in noncommunicable disease burden reflects demographic change, improved survival, and expanded diagnostic practices. Contemporary global health analyses increasingly incorporate adjustments for age structure and evolving definitions, reflecting similar methodological considerations.

Second, underlying biological risk has not uniformly increased across major conditions. Although more individuals live with and die from chronic diseases, the probability of death at a given age has declined for several major conditions, particularly cardiovascular disease and chronic obstructive pulmonary disease. This pattern reflects advances in prevention, early detection, and treatment.

Third, demographic change remains a dominant driver of global disease patterns. Population ageing and growth are the principal contributors to rising absolute case counts, as the proportion of individuals aged ≥65 years continues to increase globally.

Fourth, evolving diagnostic criteria have expanded the number of individuals classified as having chronic conditions. While this improves early detection and prevention, it complicates temporal comparisons and may contribute to overdiagnosis, particularly in older populations.

Fifth, the growing burden of chronic disease in ageing populations underscores the need for more individualised, age-appropriate approaches to care. Despite representing most patients with chronic conditions, older adults remain underrepresented in clinical trials. Greater emphasis on risk-based, patient-centred decision-making is therefore required.

Sixth, despite accounting for demographic and diagnostic effects, the global burden of noncommunicable diseases remains substantial and unevenly distributed. These conditions account for approximately three-quarters of deaths worldwide, with a disproportionate impact on low- and middle-income countries, highlighting the importance of addressing both metabolic and environmental risk factors.

Finally, the concept of a uniform, ever-expanding ‘epidemic’ of chronic disease is overly simplistic. Contemporary patterns are better understood as the result of interacting demographic, diagnostic, and risk-factor dynamics. The central challenge for public health is therefore not to reduce diagnostic vigilance, but to ensure that epidemiological interpretation, clinical decision-making, and policy responses are aligned with the realities of ageing populations.

Importantly, these findings should not be interpreted as a critique of advances in diagnosis or disease surveillance. Improved detection and earlier intervention have contributed substantially to reductions in age-specific mortality and improved outcomes. Rather, the present analysis highlights the need to interpret increases in disease prevalence within their broader demographic and epidemiological context.

Accordingly, these findings should be understood as descriptive and hypothesis-generating, rather than causal.

Future global health assessments should routinely distinguish between demographic expansion and changes in age-specific risk to avoid misinterpretation of disease trends.
